# Lockdown and visual hallucinations in older people: a community perspective

**DOI:** 10.1192/bjo.2021.348

**Published:** 2021-06-18

**Authors:** Rim Roufael

**Affiliations:** Health Education and Improvement Wales (HEIW)

## Abstract

**Objective:**

After COVID-19 was declared as a pandemic, different countries have enforced lockdowns, and shielding to mitigate the spread of the virus as preventing loss of lives was the priority.

Our aim is to look for possible explanations for increased rates of visual hallucinations presented to Community Mental Health Teams for Older People during the period of lockdown.

**Case report:**

A review of clinical cases presenting with new onset visual hallucinations to the Community Mental Health Teams for Older People during the lockdown period in 2020 was summarised in two case scenarios. One scenario represents cases with known background of dementia, while the other scenario represents new referrals during the lockdown period with no known psychiatric background. In those cases, the visual hallucinations started during lockdown with no clear cause, did not respond to psychotropic medications, physical health investigations were all normal and hallucinations improved markedly with the end of the lockdown and social isolation.

**Discussion:**

From clinical practice point of view, during the period of lockdown in the COVID-19 pandemic, visual hallucinations has been one of the commonest presentations reported to the Community Mental Health Teams for Older People. Families were calling frequently reporting that their loved ones were “seeing things”. Possible underlying causes include: social isolation, sensory and perceptual deprivation, visual impairment and Charles Bonnet syndrome, lack of cognitive stimulation activities with progress of dementia, superimposed delirium, in addition to depression secondary to loneliness, reduction in community support, increased alcohol consumption and negative effects of repeated media consumption.

**Conclusion:**

There has been a marked increase in reporting visual hallucinations in the shielding older people population in the community during the period of lockdown in the COVID-19 pandemic. This shielded population was not exposed to COVID-19, so it didn't give an explanation to this new phenomenon. Though there are multiple possible causative factors, the effect of the lockdown itself with its resultant social isolation and sensory deprivation remains to be the most significant. Shielding the older people population throughout the COVID-19 pandemic came as an essential measure as the physical safety and preventing loss of lives was the priority; however the lockdown had significant negative effects on the mental health of the shielding population. It remains unclear if those negative effects are going to be reversible in the future, resulting in poor quality of life.

